# Protective Effects of Cord Blood Serum (CBS) on Retinal Pigment Epithelium (ARPE-19) and Retinal Photoreceptor-like (661W) Cell Line Viability Under In Vitro Oxidative Stress

**DOI:** 10.3390/biom16010131

**Published:** 2026-01-12

**Authors:** Ilenia Motta, Francesca Corsi, Ilaria Piano, Silvia Bisti, Elisa Bergantin, Marina Buzzi, Maria Claudia Gargini, Piera Versura

**Affiliations:** 1 Alma Mater Institute on Healthy Planet, Alma Mater Studiorum University of Bologna, Via Massarenti 11, 40138 Bologna, Italy; ilenia.motta2@unibo.it; 2 Department of Pharmacy, University of Pisa, 56126 Pisa, Italy; francesca.corsi@farm.unipi.it (F.C.); ilaria.piano@unipi.it (I.P.); maria.gargini@unipi.it (M.C.G.); 3 National Institute of Biostructure and Biosystem (INBB), V.le Medaglie D’Oro 305, 00136 Roma, Italy; s.bisti@team.it; 4 IRCCS Azienda Ospedaliero–Universitaria di Bologna, Via Massarenti 9, 40138 Bologna, Italy; elisa.bergantin@aosp.bo.it (E.B.); marina.buzzi@aosp.bo.it (M.B.); 5 Ophtalmology Unit, Department of Medical and Surgical Sciences (DIMEC), Alma Mater Studiorum University of Bologna, Via Palagi 9, 40138 Bologna, Italy

**Keywords:** neuroprotection, cord blood serum, brain-derived neurotrophic factor (BDNF), retina pigment epithelium (ARPE-19) cells, retina photoreceptor-like (661W) cells, oxidative stress

## Abstract

Neuroprotection represents a promising approach for mitigating retinal degeneration. Cord blood serum (CBS), rich in trophic factors such as the brain-derived neurotrophic factor (BDNF), has shown therapeutic potential for ocular surface diseases; however, its role in retinal neuroprotection remains underexplored. This study evaluates the protective effects of CBS on retinal pigment epithelium (ARPE-19) and photoreceptor-like (661W) cells exposed to oxidative stress. Cells were cultured in media supplemented with fetal bovine serum (FBS) or CBS with either high (CBS-H) or low (CBS-L) BDNF content. Oxidative stress was induced using hydrogen peroxide (H_2_O_2_), and cell viability was measured via an MTS assay. ZO-1 expression was analyzed in ARPE-19 cells to assess tight junction integrity, while mitochondrial function in 661W cells was examined using MitoRed staining. TrkB receptor involvement was investigated using the inhibitor K252a and Western blot analysis. CBS significantly improved cell viability under oxidative conditions. CBS-H increased ZO-1 expression in ARPE-19 cells, indicating preserved epithelial integrity. In 661W cells, CBS maintained mitochondrial integrity and enhanced TrkB phosphorylation, while TrkB inhibition reduced its protective effect. These findings indicate that CBS confers neuroprotection through BDNF-TrkB signaling together with other trophic factors, supporting its potential as a multifactorial therapeutic strategy for retinal degeneration that deserves further exploration.

## 1. Introduction

Neuroprotection is an increasingly recognized current concept in the treatment of neurodegenerative diseases [1,2,3,4,5,6], and several approaches are under study with different mechanisms of action, focused on the delay of neuron damage progression [7,8,9,10,11].

Preparations from cord blood (CB) in various forms such as a serum (CBS) or platelet-rich plasma (CB-PRP) have become increasingly popular in recent years [12,13,14]. These include CB-based eye drops for the treatment of ocular surface disorders and stem cell-based products for regenerating injured corneal, retinal, and optic nerve tissues [15]. It is recognized that CB is rich with trophic factors whose content might vary according to several unpredictable parameters probably during gestation and labor [16,17]. CBS has been used as eye drops for pathologies related to cornea but the possibility that such a mixture of factors might be useful, even in retinal disease, exists and evidence in in vitro and in vivo models suggests to further explore the potentiality of its application as a neuroprotective agent [18,19,20,21].

The retinal pigment epithelium (RPE) is a crucial component of the retina that performs essential functions in visual processing, enabling photoreceptors to detect light. RPE cells are highly specialized epithelial cells with their apical surface in close proximity to the outer segments of photoreceptors and their basal surface anchored to Bruch’s membrane. These cells are essential for the daily renewal of photoreceptors and for supporting the nutrition and function of choriocapillaris. RPE cells phagocytose photoreceptor outer segments (POS) to facilitate the ongoing renewal of photoreceptor cells. However, the persistence of photooxidative stress induces an accumulation of reactive oxygen species (ROS). In physiological conditions, the antioxidants present in RPE cells effectively neutralize oxygen radicals to maintain homeostasis. In contrast, RPE cells affected by age-related macular degeneration (AMD) exhibit elevated levels of apoptosis and autophagy, which increases the oxidative stress burden. This ongoing oxidative stress disrupts phagocytosis and autophagy in RPE cells, leading to increased protein aggregation and activation of inflammatory vesicles [22,23,24].

The purpose of this study was to focus on RPE cells and retinal photoreceptor-like cells (661W), which are a district cell type, to evaluate the efficacy of CBS in the protection of cells directly involved in the retinal degenerative process, in an in vitro model of oxidative stress induction.

## 2. Materials and Methods

### 2.1. Cell Culture and Reagents

Retinal pigment epithelial cell line (ARPE-19) was purchased from ATCC and cultured in Dulbecco’s Modified Eagle Medium/Nutrient Mixture F12 (DMEM/F12, Gibco, London, UK) enriched with 10% fetal bovine serum (FBS) and 1% penicillin/streptomycin solution in a humidified incubator (5% CO_2_, 37 °C).

The 661W photoreceptor cells were supplied by Dr. Muayyad Al-Ubaidi (University of Oklahoma Health Sciences Center) [25] and cultured in DMEM—high glucose with 10% FBS and 1% penicillin/streptomycin solution in a humidified incubator (5% CO_2_, 37 °C).

The material used for cell cultures was purchased from Sigma-Aldrich (Merck, Darmstadt, Germany).

### 2.2. Product—CBS Preparation and Dosage

The study was performed following the principles of the Declaration of Helsinki. The use of the CBS was approved by the local Ethical Committee for the assessment of the product for research purposes (128/2017/U/Sper).

CB units were collected at the time of delivery after compilation of a donor selection questionnaire and signing informed consent; all steps from the recruitment to the processing and registration of CB were performed according to standard operating procedures and guidelines issued by the Foundation for the Accreditation of Cellular Therapy (FACT). CB was collected from spontaneous term births free of complications (≥37th week of pregnancy) and Cesarean births decided by trained and qualified health personnel. CB collection for research purposes was performed from ex utero placenta vessels, and transferred in a 9 mL Vacumtube (Biomed Device, Modena, Italy) without any anticoagulant. The medium volume of collected samples was 7 ± 1.5 mL. For further processing, the samples were sent to the Processing Facility (PF) laboratory of the CB Bank where it went through a series of checks and tests to establish the blood characteristics and its suitability for preservation and therapeutic use. Maternal infectious disease marker (HIV, HCV, HBV Treponema pallidum, CMV, Toxoplasmosis, and HTLVI/II) evaluations were performed. Suitable samples were centrifuged at 2800× *g* for 10 min to obtain the serum (CBS) and then transferred in sterile tubes under laminar flow hood and stored at −80 °C. CBS samples (n = 25) were tested for the level of BDNF (brain-derived neurotrophic factor) content with an ELISA kit (R&D Systems, Bio-techne, Minneapolis, MN, USA), and subsequently pooled to obtain aliquots containing either high-level or low-level BDNF to be used in the experiments. The analysis was repeated after heat treatment (56 °C for 20 min) for complement inactivation.

### 2.3. Stress Model—Oxidative Stress

FBS or CBS (with a high or low level of BDNF) were added to the growth medium at 10%. Hydrogen peroxide (H_2_O_2_) at 30% (Sigma-Aldrich, Merck, Darmstadt, Germany) was used to induce oxidative stress and was diluted in sterilized water at 9.88 mM, before preparation of working solutions in a serum-free medium for cell treatments.

ARPE-19: briefly, cells were seeded in 96-well plate at 2.5 × 10^4^ cells/well in 100 μL growth medium. After 24 h, the cell medium was changed with a growth medium containing 10% FBS or CBS and after another 24 h cells were treated with H_2_O_2_ 500 μM. Cell viability assay was performed after 24 h.

661W: cells were seeded in 96-well plate at 1 × 10^4^ cells/well in 100 μL growth medium. After 24 h, cells were put into starvation for 4 h and then the medium was changed with a growth medium containing 10% FBS or CBS and after another 24 h cells were treated with H_2_O_2_ 500 μM for 3 h. Cell viability assay was performed after 24 h.

### 2.4. Tropomyosin Receptor Kinase (Trk) Inhibition

For the inhibition of Trk receptors, cells were treated with 500 nM of K252a (K1639, Sigma-Aldrich, Merck, Darmstadt, Germany) for 1 h. Then, the culture medium was changed with FBS or CBS at 10% for 24 h, followed by the addition of H_2_O_2_ 500 μM for 24 h. CellTiter 96 AQueous One Solution Cell Proliferation assay (Promega, Madison, WI, USA) was then performed for the analysis of cell viability.

### 2.5. Viability Assay

Cell viability was assessed by using CellTiter 96 AQueous One Solution Cell Proliferation kit (Promega, Madison, WI, USA) following the manufacturer’s instructions. Absorbance was read at optical density (O.D.) 490 nm by microplate reader.

ARPE-19: cell survival rate was normalized to ARPE-19 grown in medium without serum.

661W: cell survival rate was normalized to 661W grown in medium with 10% FBS.

### 2.6. Immunofluorescence

Immunofluorescence was assessed to detect the expression of Zonula Occludens-1 (ZO-1) protein in ARPE-19 cells. This is an essential structural protein, signaling the membrane integrity of ARPE-19 [26]. Briefly, cells were seeded at 1 × 10^5^ on glass slides in the growth medium enriched with 10% FBS or CBS. After 24 h, cells were treated with H_2_O_2_ 500 μM according to the experimental scheme. After the treatment, cells were washed with PBS and fixed with 4% paraformaldehyde (PFA) for 10 min at room temperature. The blocking of non-specific binding sites was performed with bovine serum albumin (BSA) 1% in PBS for 30 min at room temperature. After blocking, cells were incubated with ZO-1 (1:500, Thermo Fisher Scientific, Waltham, MA, USA) for 1 h at 37 °C. Samples were then washed with PBS and incubated with anti-rabbit Alexa Fluor 546 (1:200, Thermo Fisher Scientific, Waltham, MA, USA) secondary antibody for 1 h at 37 °C in the dark. After washing with PBS, nuclei were counterstained with DAPI. Images were acquired by a Leica DMI4000 B inverted fluorescence microscope (Leica Microsystems, Wetzlar, Germany). Quantification of ZO-1 fluorescence intensity was performed with ImageJ-8 software on digitalized images randomly acquired at 20× magnification, and a minimum of 3 fields were examined for each sample.

The mitochondrial membrane integrity was assessed to evaluate the 661W cell function preservation, as suggested by Corsi et al. [27]. To evaluate the efficacy of CBS against H_2_O_2_ damage (500 μM for 3 h) in 661W, cells were seeded into an 8-well chamber slide at a density of 10^4^ cells/well, then cells were treated according to the experimental scheme. After treatment, cells were stained by adding 50 nM MitoRed kit (Sigma-Aldrich, Merck, Darmstadt, Germany) diluted in DMEM for 1 h at 37 °C, before fixation with 2% PFA for 15 min, followed by three washes with PBS. After washing, cell nuclei were stained with DAPI (Sigma-Aldrich, Merck, Darmstadt, Germany) 1:5000 in PBS. Coverslips were mounted with Vectashield on a microscope support and imaged with a Nikon confocal microscope, with a 40× (water) objective.

### 2.7. Gene Expression Analysis

Total RNA extraction was performed by using TRIreagent (TRIzol reagent; Thermo Fisher Scientific, Waltham, MA, USA) according to the manufacturer’s instructions. Reverse transcription was performed from 1 µg of total RNA in 20 µL reaction volume using iScript^TM^ cDNA synthesis kit (BioRad Laboratories, Hercules, CA, USA). Real-time PCR was carried out to analyze ZO-1 gene expression in ARPE-19 in a CFX Connect Real-Time PCR Detection System (BioRad Laboratories, Hercules, CA, USA) using the SYBR green mix (SsoAdvancedTM Universal Sybr Green Supermix; BioRad Laboratories, Hercules, CA, USA). Primer sequences were designed using the NCBI BLAST tool (https://blast.ncbi.nlm.nih.gov/Blast.cgi) and purchased from Merck Group (Darmstadt, Germany) (Table 1). Each assay was performed at least in triplicate and the target gene expression was normalized to glyceraldehyde 3-phosphate dehydrogenase (GAPDH). Gene expression levels were determined by the comparative 2^−ΔΔCt^ method and expressed as fold changes relative to untreated control cultured with growth medium without serum [28].

### 2.8. Western Blot

Cells were seeded in a 6-well plate at a density of 3 × 10^5^ cells. After the protocol treatment both cell lines were lysed by adding 200 µL of RIPA buffer (150 mM NaCl, 50 mM Tris-HCl pH 8, 1% Igepal, 0.5% Na-deoxycholate, 0.1% SDS; and protease inhibitors- 1 µM Orthovanadate and 0.1 mg/mL PMSF). The procedure of lysis, quantification, electrophoretic run of protein samples, and antibodies incubation and analysis was described previously [21]. Briefly, 30 μg of each cell protein extract was mixed with the Laemmli 4X solution and resolved by electrophoresis SDS-PAGE, using precast stain-free gel, and after activation, the separated proteins were transferred to PVDF membranes. The membranes were incubated with the primary antibodies t-TrkB 1:500 (Santa Cruz Biotechnology, Dallas, TX, USA) and p-TrkB 1:500 (Thermo Fisher Scientific, Waltham, MA, USA) and then their specific secondary antibodies. Analysis of the densitometry was undertaken using Bio-Rad ImageLab 6.1 software. Original western blots can be found in the Supplementary Materials.

### 2.9. Statistics

Each experiment was executed at least in triplicate. Results were analyzed by GraphPad Prism 8 statistical software (GraphPad Software Inc.) and expressed as mean ± standard deviation. Statistical analysis was performed using One-Way ANOVA test followed by Turkey’s multiple comparisons test. Results were considered statistically significant at the 95% confidence level (*p* value < 0.05 was considered as significant).

## 3. Results

### 3.1. BDNF Levels in CBS

The levels of BDNF in CBS single samples evidenced a high interindividual biological variability. The samples were grouped to achieve varying levels of BDNF content, with aliquots containing either a low level (6–10,000 pg/mL) or a high level (20–23,000 pg/mL) of BDNF. These different products were used and compared in the subsequent experiments.

### 3.2. CBS Increase Cell Viability of ARPE-19 and 661W Exposed to Oxidative Stress

**Figure 1:**
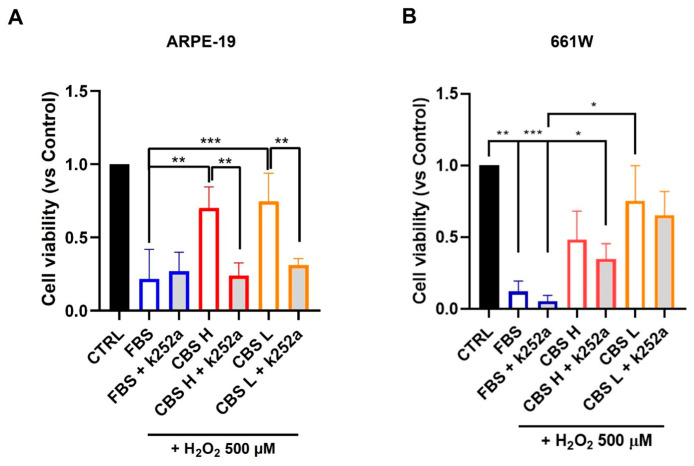
CBS improves ARPE-19 and 661W cell viability under oxidative stress. ARPE-19 (**A**) and 661W (**B**) cells were cultured with FBS or CBS with high (CBS H) and low (CBS L) concentrations of BDNF and exposed to H_2_O_2_ 500 μM. The same conditions were also tested in the presence of k252a (500 nM). Cell viability was assessed by MTS assay to investigate protective effects of CBS, showing a significant increase in cell survival compared to cells cultured in FBS. For ARPE-19, MTS data were normalized to cells grown in medium without serum, while for 661W MTS data were normalized to cells grown in medium with 10% FBS. n = 3 for each condition. Statistical analysis was performed using One-Way ANOVA test followed by Turkey’s multiple comparisons test. * *p* < 0.05; ** *p* < 0.01; *** *p* < 0.001.

The protective effect of CBS was analyzed in ARPE-19 and 661W cells under oxidative stress. We conducted a preliminary analysis to determine if CBS administration affects cell viability. In ARPE-19 cells we observed no significant differences in growth between FBS and CBS. Meanwhile, in the 661W cells we obtained a slight reduction in cell viability with CBS L, probably due to a lower concentration of growth factor for a neuronal cellular line (Supplementary Materials Figure S1). Therefore, ARPE-19 and 661W cells were cultured with FBS or CBS with a high (CBS H) and low (CBS L) concentration of BDNF and their viability was tested after treatment with H_2_O_2_. CBS improved cell viability under oxidative stress, as indicated by the MTS assay, which shows significant differences between FBS and CBS (Figure 1). Considering the presence of neurotrophins in CBS, we investigated whether inhibiting the Trk receptor would reduce the efficacy of CBS. Therefore, we treated ARPE-19 and 661W cells with the Trk inhibitor K252a (500 nM) for 1 h. The presence of K252a resulted in a significant decrease in the protective effect of CBS, corresponding to a lower cell viability under oxidative stress (Figure 1).

### 3.3. Effects of CBS on ZO-1 Expression in ARPE-19 Under Oxidative Stress

**Figure 2:**
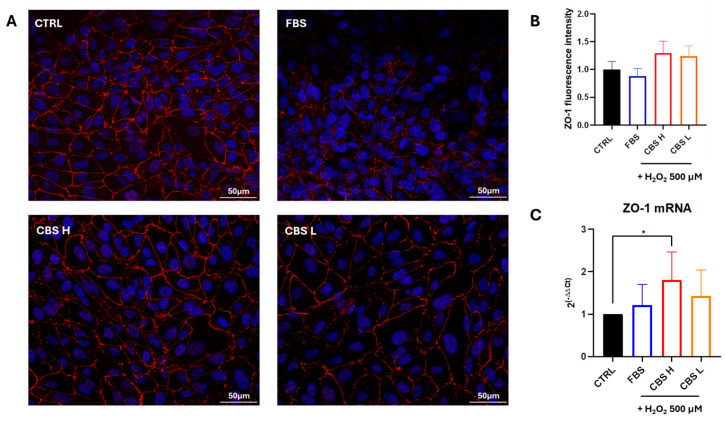
ZO-1 expression improves in ARPE-19 cultured with CBS. ARPE-19 were cultured with FBS or CBS with high or low concentration of BDNF and exposed to H_2_O_2_ 500 μM. Immunofluorescence shows an enhanced expression of ZO-1 protein (in red) in CBS H and CBS L compared to FBS (**A**,**B**); scale bars 50 µm. Real-time PCR analysis presents an increased expression of ZO-1 in ARPE-19 cultured with CBS H (**C**). Real-time data are reported as fold changes relative to untreated control, in DMEM without serum (CTRL), and are expressed as mean ± standard deviation of three independent experiments. Values are normalized to control (set as 1). n = 3 for each condition. Statistical analysis was performed by One-Way ANOVA test followed by Turkey’s multiple comparisons test. * *p* < 0.05.

To evaluate the improvement of the integrity of ARPE-19 cells, ZO-1 expression was evaluated. ARPE-19 cells were cultured with FBS or CBS with a high and low level of BDNF and their viability was tested after treatment with H_2_O_2_ for 24 h. We observed an improvement of ZO-1 protein expression in cells cultured with CBS (Figure 2A,B). Moreover, a significant increase in the expression of ZO-1 mRNA was detected in ARPE-19 grown with CBS H in comparison to serum-free control cells (Figure 2C), confirming the protective effects of CBS in ARPE-19.

### 3.4. Effects of CBS on Mitochondrial Membrane Potential in 661W Under Oxidative Stress

**Figure 3:**
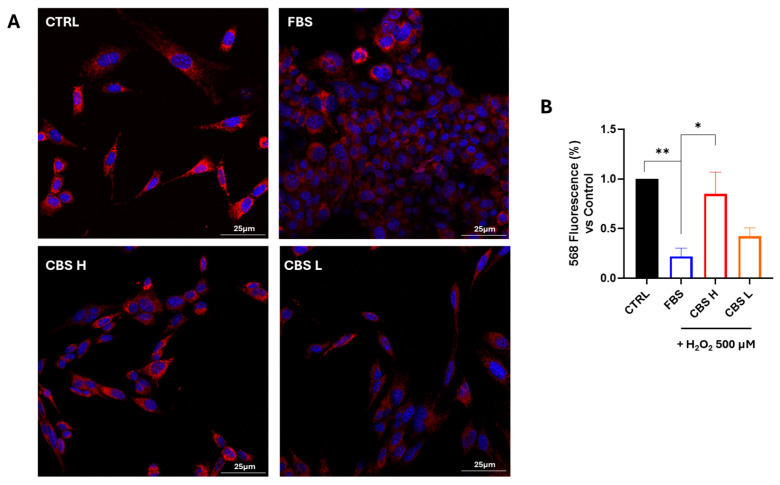
CBS improves mitochondrial viability in 661W cells under oxidative stress. 661W cells were cultured with FBS or CBS with high (CBS H) and low (CBS L) concentrations and exposed to H_2_O_2_ 500 μM. (**A**) Images show the staining for a mitochondria-specific marker (MitoRed, in red). Cell nuclei (DAPI) are stained blue. Scale bars 25 μm. (**B**) Quantification of fluorescence intensity of cells normalized with CTRL. Values are normalized to control (set as 1). n = 3 for each condition. Statistical analysis was performed using One-Way ANOVA test followed by Turkey’s multiple comparisons test. * *p* < 0.05; ** *p* < 0.01.

Next, we tested whether CBS could mitigate H_2_O_2_-mediated 661W cell death while preserving the cell membrane of mitochondria undamaged. To do this, we stained cells with MitoRed, a specific dye of undamaged cell membrane of mitochondria. In Figure 3A, representative images, acquired by microscopy, show how the membrane staining of CBS-treated mitochondria is higher than that of cells stressed with hydrogen peroxide. The results obtained from confocal image acquisition were confirmed by MitoRed fluorescence quantification, summarized in Figure 3B. The expression of MitoRed increased significantly in 661W cells after CBS H pretreatment, thus validating its protective effect on mitochondria.

### 3.5. Alterations in TrkB and Phosphorylated p-TrkB Levels in ARPE-19 and 661W Cells

**Figure 4:**
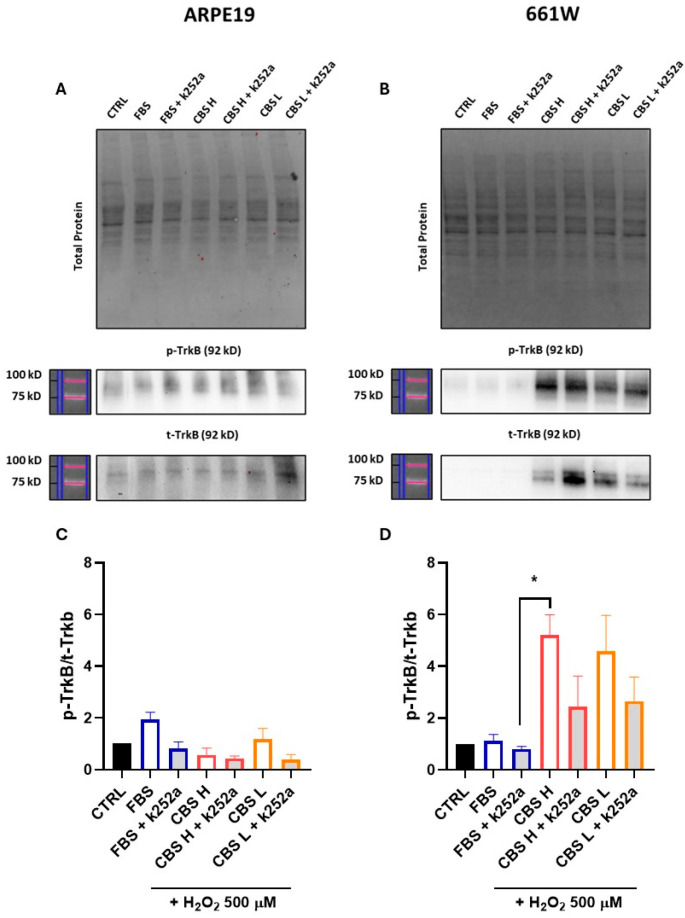
Treatment with CBS activated TrkB. (**A**,**B**) Representative example of Western blot obtained by loading all samples under analysis; (**C**) bar graph represents p-TrkB/t-TrkB ratio in ARPE-19; and (**D**) bar graph represents p-TrkB/t-TrkB ratio in 661W. n = 3 for each condition. Uncropped Western blots for Figure 4 are found in Supplementary Figures S2 and S3. Values are normalized to control (set as 1). Statistical analysis was performed using One-Way ANOVA. * *p* < 0.05 (661W: FBS + k252a vs. CBS H).

To investigate whether activation or inhibition of TrkB drives the efficacy of CBS treatment, we performed protein analysis of TrkB and phosphorylated TrkB (p-TrkB), an activated form of TrkB, in samples from both ARPE-19 and 661W treated with CBS H and L, and from cells treated with CBS and the Trk receptor inhibitor (K252a). An increase in the ratio of p-TrkB/t-TrkB was observed in both H and L CBS-treated 661W cells, compared with treatment to both FBS-cultured cells and cells treated with the specific TrkB inhibitor, thus providing a possible pathway of CBS efficacy on these cells. Also, in ARPE-19, the ratio of p-TrkB/t-TrkB treated with CBS is higher than those treated with the inhibitor (Figure 4).

## 4. Discussion

Data from this study demonstrate that the CBS supply protects ARPE-19 and 661W cells from oxidative stress damage in an in vitro model. CBS reduces the mortality rate of both cell lines by supplying both high and low levels of BDNF. CBS supply also preserves the function of ARPE-19 cells, as supported by the expression of ZO-1, a tight junction protein crucial for cell integrity [25], and of 661W cells, as indicated by the maintenance of mitochondrial membrane integrity [26]. Finally, the data suggests that BDNF is not the sole contributor to the protective mechanism, supporting the role of CBS as a whole, since this product contains a pool of several neurotrophins beyond BDNF.

BDNF was considered as a reference neurotrophic factor, based on previous studies demonstrating its neuroprotective role and its ability to reach the retina following topical administration in animal models of retinal degeneration [29]. However, CBS contains many growth factors, including neurotrophins, so other trophic elements, not considered in the present work, would play a role in the protection mechanisms. Analysis of BDNF levels in CBS single samples revealed significant interindividual biological variability, as demonstrated in previous reports [20]. With the aim to understand the impact of different contributions of BDNF in experiments, different pools were constituted and named low BDNF (BDNF L) and high BDNF (BDNF H). The BDNF H supply (20–23,000 pg/mL in the original source) appeared to be more effective in preserving function in both cell lines. Papers published in the literature report BDNF as a protective factor for retinal cells. Lambuk et al. reviewed the crucial role of BDNF in the survival of retinal ganglion cells (RGCs). The exogenous application of BDNF in experimental models of glaucoma showed the improvement of RGCs survival and reduction in their degeneration, however its effects are short-lived. Repeated exposure can lead to decreased responsiveness and desensitization of TrkB activation. Additionally, delivering BDNF to target sites is challenging due to its instability. BDNF gene delivery using recombinant adeno-associated viruses has shown promise in increasing BDNF concentrations in the retina and supporting RGCs survival. This approach could effectively ensure BDNF delivery to the target site of RGCs. However, it does not elevate BDNF levels in the retina or protect RGCs in models of glaucoma. Although preclinical studies have demonstrated neuroprotective effects of BDNF, its clinical application still requires further investigation [30].

In the present paper the effect of natural BDNF delivered was estimated, with possible differences in target, affinity, and kinetics with respect to the recombinant one, so that a comparison in terms of levels of efficacy is not feasible at present.

The protective effects of CBS supply from oxidative stress damage were shown by evaluating ZO-1 expression in ARPE-19. ZO-1 plays a crucial role in ARPE-19 cells, acting as a key tight junction protein that maintains RPE integrity. It facilitates the formation of cell–cell junctions, regulating cell adhesion and barrier functions. Disruption of ZO-1 can compromise barrier function and alter cellular dynamics, which may have implications for retinal health, potentially contributing to retinal disorders and impairing the protective role of the RPE. Overall, ZO-1 is essential for preserving the structural and functional properties of the RPE [26,31].

To assess the ability of CBS to protect 661W cells from H_2_O_2_-induced damage, the metabolic state of the cells was evaluated by assessing mitochondrial damage. Nerve cells, particularly photoreceptors, are known to have high cellular metabolism [32], necessary for the performance of their function, so neuroprotective treatments that act by keeping the mitochondria functional ensure greater functionality and survival of the cells themselves. In the present work we show that treatment with CBS, and with CBS H, is able to preserve mitochondria from oxidative stress, with the mechanism likely mediated, at least in part, by BDNF activation of the TrkB receptor. Experimental evidence shows that, at the neuronal level, increased oxidative stress causes a reduction in the levels in the proportion of the TrkB receptor that is phosphorylated and consequently activated to induce an antioxidant and anti-apoptotic biological response [33]. Also in the present study, similar 661W cells damaged with hydrogen peroxide showed a reduction in the p-Trkb/t-Trkb ratio, whereas treatment with CBS has the capacity to increase the phosphorylated portion of the receptor. This effect, induced by CBS treatment, could be the pathway by which cells trigger a protective mechanism against oxidative damage. In the case of retinal pigment epithelium cells, while CBS treatment offered some protective effect, this did not correlate with activation of the TrkB pathway, as phosphorylation levels were not modulated by CBS treatment. This lack of activation could be due to the low levels of the receptor in epithelial cells compared with cells of nerve origin, so that in the case of ARPE-19 the protective effect is to be sought in other mechanisms or probably in a synergic action of several factors contained in the complex CBS pool. To corroborate that the protective effect in 661W nerve cells depends on activation of the Trkb pathway, we inhibited Trkb with K252a and performed additional experiments under the same stress conditions. We observed a significant reduction in the protective effects of CBS, suggesting that inhibiting TrkB impairs the neurotrophic effects of CBS on cell protection in the presence of damage.

## 5. Conclusions

This study provides initial evidence that cord blood serum (CBS), as a natural source of neurotrophins, can protect retinal cells from oxidative stress. CBS preserved mitochondrial integrity in photoreceptor-like 661W cells and maintained ZO-1 expression in ARPE-19 cells, suggesting distinct but complementary protective mechanisms. Beyond the contribution of BDNF, the synergistic action of multiple growth factors appears central to these effects. Although limited to in vitro models, these findings encourage further in vivo studies to clarify the role of individual factors and to explore CBS-based formulations as potential therapeutic options for retinal diseases.

## 6. Patents

A patent, “Blood serum for use in the treatment of neurodegenerative ophthalmologic pathologies”, covering the topic of this manuscript, was filed on 15 June 2017 and is owned by University of Bologna, IRCCS AOU BO and University of L’Aquila. PV, MB, and SB are among the inventors of the patent and participated to this research without any commercial involvement. Other authors have no competing interest.

## Figures and Tables

**Table 1:** List of primer sequences used for gene expression analysis. FWD, forward; REV, reverse; ZO-1, zonula occludens-1; GAPDH, glyceraldehyde 3-phosphate dehydrogenase.

Gene	Primer Sequence
ZO-1	FWD: 5′TCACCTACCACCTCGTCGTC 3′ REV: 5′ ATGAGCACTGCCCACCCAT 3′
GAPDH	FWD: 5′AATGGGCAGCCGTTAGGAAA 3′ REV: 5′ AGGAGAAATCGGGCCAGCTA3′

## Data Availability

The data generated in this study are not publicly available due to the use of regulated human-derived biological material and the experimental nature of the datasets, which require controlled access and contextual interpretation in accordance with institutional regulations. Data access may be considered by the corresponding author upon reasonable request, in the context of scientific comparison or collaborative research with groups working on related topics.

## Supplementary Materials

The following supporting information can be downloaded at: https://www.mdpi.com/article/10.3390/biom16010131/s1, Figure S1: Preliminary analysis of cell viability following CBS administration; Figure S2: Representative uncropped Western blots showing total protein (A), p-TrkB (B), and t-TrkB (C) protein expression in 661W cells; Figure S3: Representative uncropped Western blots showing total protein (A), p-TrkB (B), and t-TrkB (C) protein expression in ARPE-19 cells.
