# Supplementary material for: Protective Effects of Cord Blood Serum (CBS) on Retinal Pigment Epithelium (ARPE-19) and Retinal Photoreceptor-like (661W) Cell Line Viability Under In Vitro Oxidative Stress
Source: Biomolecules. 2026 Jan 12;16(1):131. doi: 10.3390/biom16010131 (PMC12838533; doi:10.3390/biom16010131)
Supplement: Supplementary file 1 [file biomolecules-16-00131-s001.zip › biomolecules-3897086-supplementary.pdf]

Supplementary Material

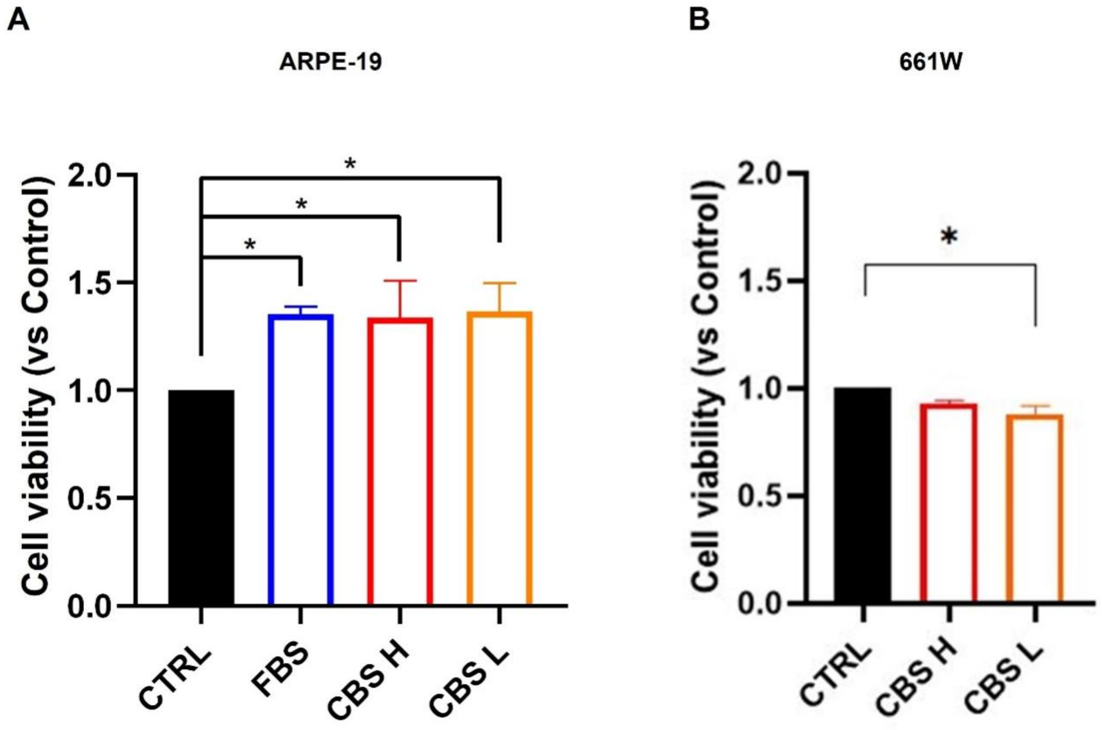

Figure S1. Preliminary analysis of cell viability following CBS administration. In ARPE-19, no significant differences in cell growth were observed between cells cultured with FBS and those cultured with CBS, indicating that CBS does not adversely affect or improve cell viability. In 661W cells, a slight reduction in cell viability with CBS L was observed, probably due to a lower concentration of growth factor for a neuronal cell line. Statistical analysis was performed using One-Way ANOVA test followed by Turkey's multiple comparisons test. *$p \leq 0.05$ (ARPE-19: CTRL vs FBS, CTRL vs CBSH, CTRL vs CBSL; 661W: CTRL vs CBSL).

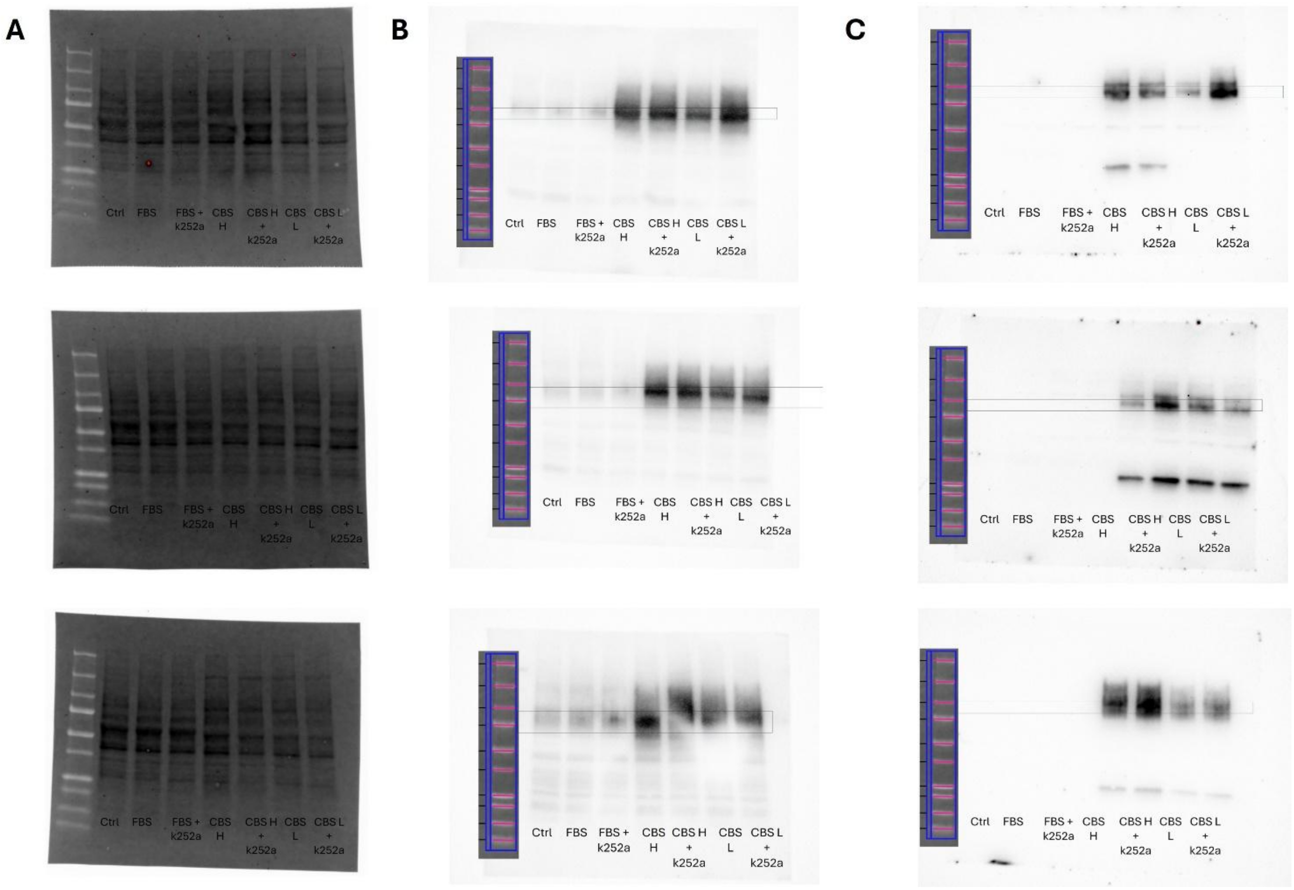

Figure S2. Representative uncropped western blots showing Total protein (A), p-TrkB (B), and t-TrkB (C) protein expression in 661W cells.

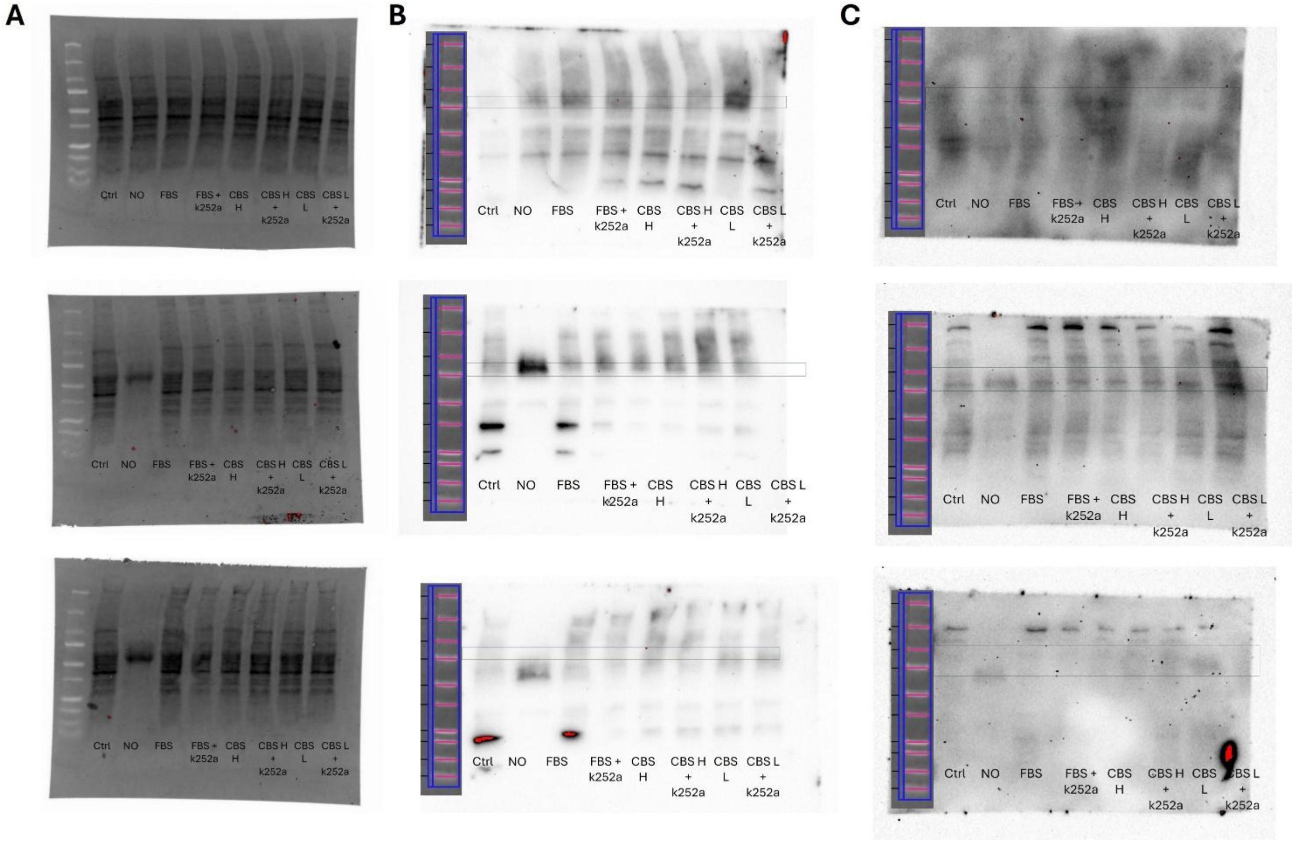

Figure S3. Representative uncropped western blots showing Total protein (A), p-TrkB (B), and t-TrkB (C) protein expression in ARPE-19 cells.
